# Mint3/Apba3 depletion ameliorates severe murine influenza pneumonia and macrophage cytokine production in response to the influenza virus

**DOI:** 10.1038/srep37815

**Published:** 2016-11-24

**Authors:** Takayuki Uematsu, Tomoko Fujita, Hiroki J. Nakaoka, Toshiro Hara, Noritada Kobayashi, Yoshinori Murakami, Motoharu Seiki, Takeharu Sakamoto

**Affiliations:** 1 Department of Biomedical Research, Biomedical Laboratory, Kitasato University Medical Center, Arai, Kitamoto, Saitama Japan; 2 Innovation Laboratory, NEC Solution Innovators, Ltd., Kashiwanoha, Kashiwa, Chiba Japan; 3 Division of Molecular Pathology, Institute of Medical Science, The University of Tokyo, Shirokanedai, Minato-ku, Tokyo Japan; 4 Division of Cancer Cell Research, Institute of Medical Science, The University of Tokyo, Shirokanedai, Minato-ku, Tokyo Japan; 5 Faculty of Medicine, Institute of Medical, Pharmaceutical and Health Sciences, Kanazawa University, Takara-machi, Kanazawa, Ishikawa Japan

**Keywords:** Stress signalling, Monocytes and macrophages, Influenza virus

## Abstract

Influenza virus (IFV) infection is a common cause of severe pneumonia. Studies have suggested that excessive activation of the host immune system including macrophages is responsible for the severe pathologies mediated by IFV infection. Here, we focused on the X11 protein family member Mint3/Apba3, known to promote ATP production via glycolysis by activating hypoxia inducible factor-1 (HIF-1) in macrophages, and examined its roles in lung pathogenesis and anti-viral defence upon IFV infection. Mint3-deficient mice exhibited improved influenza pneumonia with reduced inflammatory cytokines/chemokine levels and neutrophil infiltration in the IFV-infected lungs without alteration in viral burden, type-I interferon production, or acquired immunity. In macrophages, Mint3 depletion attenuated NF-κB signalling and the resultant cytokine/chemokine production in response to IFV infection by increasing IκBα and activating the cellular energy sensor AMPK, respectively. Thus, Mint3 might represent one of the likely therapeutic targets for the treatment of severe influenza pneumonia without affecting host anti-viral defence through suppressing macrophage cytokine/chemokine production.

**Electronic supplementary material:**

The online version of this article (doi:10.1038/srep37815) contains supplementary material, which is available to authorized users.

## Introduction

Influenza is the most prevalent viral respiratory infection during winter. Occasionally, large-scale infectious outbreaks or pandemics on a global scale have arisen throughout history, which have caused numerous deaths^1,2,3,4,5^. Primary influenza viral pneumonia (PIVP), the most severe pulmonary manifestation of influenza, is caused by the proliferation and activation of influenza virus (IFV) in the lungs with rapid progression of symptoms. Many patients with PIVP develop acute respiratory distress syndrome (ARDS) that is very difficult to treat and has a high mortality rate^6,7^. PIVP incidence was higher in past outbreaks of the pandemic H1N1 2009 virus and avian influenza A H5N1 virus infection than that in seasonal influenza infection^8,9,10,11^. Although vaccinations and anti-viral agents are effective in influenza, they are not considered a fundamental means to achieve remission in severe cases. Therefore, an effective strategy that does not rely on existing therapeutic concepts is required. Recent studies have found that excessive activation of the host innate immune system in response to the virus is a major factor that increases the severity of influenza^12,13,14,15^. At the onset of PIVP, invading viruses trigger the host innate immune system including factors such as macrophages, dendritic cells, neutrophils, and fibroblasts. These activated cells secrete inflammatory cytokines/chemokines including IL-6, TNF, CXCL1, and CXCL10^11,16^, which play a crucial role in the pathogenesis of ARDS^12,15,17,18,19^. Therefore, the inhibition of cytokine/chemokine production targeting innate immunity might be an effective treatment choice for ARDS.

Munc18–1-interacting protein 3 (Mint3), also known as amyloid-β A4 precursor protein-binding family A member 3 (Apba3), is a member of the X11 protein family^20,21^. Mint3 is expressed in most cell types and localizes to the trans-Golgi network by interacting with membrane proteins such as amyloid precursor protein and furin^22,23^. Previously, we have revealed that Mint3 activates hypoxia-inducible factor 1 (HIF-1) even in the presence of oxygen by suppressing its inhibitor, factor-inhibiting HIF-1 (FIH-1)^24^. Mint3 requires a transmembrane protease MT1-MMP/MMP14 to bind and suppress FIH-1^25,26^. Thus, Mint3-mediated HIF-1 activation is limited to MT1-MMP expressing cells such as macrophages and cancer cells^25,26,27^. Mint3-deficient mice are viable and show no apparent defect. However, Mint3-deficent macrophages exhibit a defect in ATP production via glycolysis owing to reduced HIF-1 activity^25,27,28^. Mint3-deficient macrophages also showed impaired cytokine production in response to lipopolysaccharide (LPS)^27^. Since FIH-1 also hydroxylates molecules other than HIF-1α including IκBα^29^ which inhibits the NF-κB signalling, FIH-1 suppression by Mint3 might affect inflammatory cytokine/chemokine production via not only HIF-1 but also the NFκB signalling in macrophages. Because macrophages are one of the main sources of inflammatory cytokines/chemokines, here we hypothesized that Mint3 likely played roles in influenza pneumonia and in host protection against IFV.

In this study, we demonstrate a role of Mint3 in influenza pathogenesis and immunity. Our results showed that Mint3 depletion in mice attenuated fatal influenza pneumonia through the reduction of inflammatory cytokine/chemokine production and neutrophil infiltration without affecting type-I interferon production and anti-viral acquired immunity. Further analyses revealed that Mint3 depletion attenuated NF-κB signalling in response to IFV infection in macrophages. Therefore, Mint3 inhibition might represent one of the likely targets for the treatment of severe influenza pneumonia.

## Results

### Mint3 depletion attenuates lethal influenza pneumonia in mice

**Figure 1:**
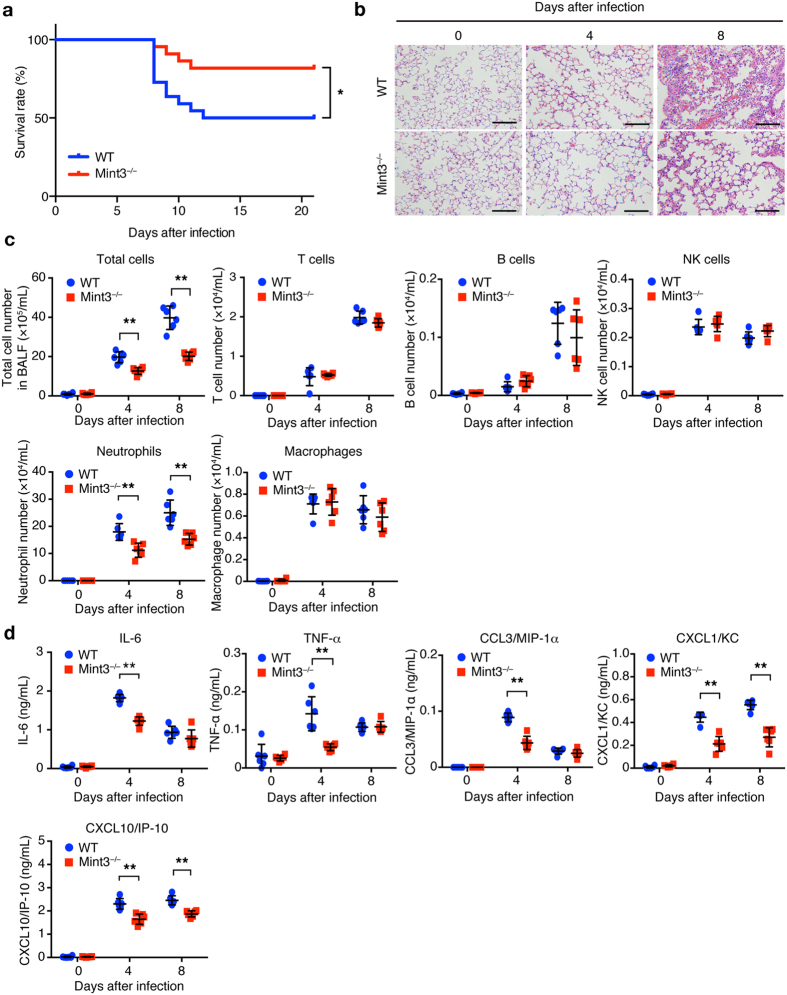
Loss of Mint3 attenuates severe influenza pneumonia. (**a**) Survival rate of mice after IFV infection. WT and Mint3^−/−^ mice (n = 22 per group) were intranasally infected with 10^4^ PFU of IFV, followed by the assessment of survival rates. *P < 0.05 by the log-rank test. (**b**) Hematoxylin and eosin staining of the lungs on days 0, 4, and 8 after IFV infection. Bar = 50 μm. (**c**,**d**) Analysis of the BALF collected from WT and Mint3^−/−^ mice (n = 6 per group) on day 0, 4, and 8 after IFV infection for infiltrated cells (**c**) and inflammatory cytokine/chemokine levels (**d**). Infiltrated cells in BALF were analysed for T cells (CD45^+^ CD3ε^+^), B cells (CD45^+^ CD19^+^), natural killer (NK) cells (CD45^+^ NK1.1^+^), neutrophils (CD45^+^ Ly-6G^+^ F4/80^−^), and macrophages (CD45^+^ Ly-6G^−^ F4/80^+^) by flow cytometry and the numbers of total and specific cell populations per lung were calculated. Cytokine/chemokine levels in BALF were measured using a multiplex assay. Data are presented as the means ± SD and are representative of two independent experiments. *P < 0.05, **P < 0.01 by the Student’s t-test.

To determine whether Mint3 contributes to immune responses during lung IFV infection, we first intranasally infected wild-type (WT: C57BL/6) and Mint3-deficient (Mint3^−/−^) mice with a lethal dose of IFV (10^4^ plaque forming units (PFU)/mouse). IFV-infected WT mice appeared visibly ill with ruffled fur and reduced oral intake 6 to 10 days after infection, whereas Mint3^−/−^ mice appeared more active than the WT mice. Consistent with their apparent activity, the final survival rate on day 21 after infection was significantly improved in Mint3^−/−^ mice (~80%) as compared with that in WT mice (~50%; Fig. 1a). Histopathological analysis of the IFV-infected lungs on days 4 and 8 revealed that lung inflammation, which was the most obvious on day 8 in WT mice, was much less severe in Mint3^−/−^ mice (Fig. 1b).

Given that inflammatory cytokines/chemokines are linked to lung damage in severe influenza pneumonia^12,13,14,17,18,19,30^, we analysed the infiltrated cells and cytokine/chemokine levels in bronchoalveolar lavage fluid (BALF) collected from the IFV-infected lungs of WT and Mint3^−/−^ mice. Consistent with the pathological data, the total number of infiltrated cells in BALF from Mint3^−/−^ mice was decreased compared to that from WT mice on days 4 and 8 after IFV infection (Fig. 1c and Supplementary Fig. S1a–c). This was attributed to the significant decrease in neutrophils that comprised the major subpopulations of infiltrated cells (Fig. 1c). In contrast, the numbers of T, B, and natural killer cells as well as macrophages were not significantly altered in BALF from the Mint3^−/−^ lungs (Fig. 1c). The levels of inflammatory cytokines and chemokines, i.e., IL-6, TNF-α, CCL3/MIP-1α, CXCL1/KC, and CXCL10/IP-10, which have been reported to contribute to lung pathology^11,12,16^, also decreased in BALF from Mint3^−/−^ mice compared to those from WT mice on day 4 after infection (Fig. 1d). In addition, the levels of CXCL1/KC and CXCL10/IP-10 in BALF from Mint3^−/−^ mice remained lower than those from WT mice on day 8 after infection. These data indicate that host Mint3 contributes to cytokine/chemokine production including the neutrophil chemoattractant CXCL1/KC at an early time point after IFV infection and influences subsequent inflammatory cell recruitment and lung pathology at a later time point. Collectively, Mint3 depletion attenuated the severe influenza pneumonia and improved host mortality in mice.

### Mint3 depletion does not compromise anti-viral protective immunity in mice

**Figure 2:**
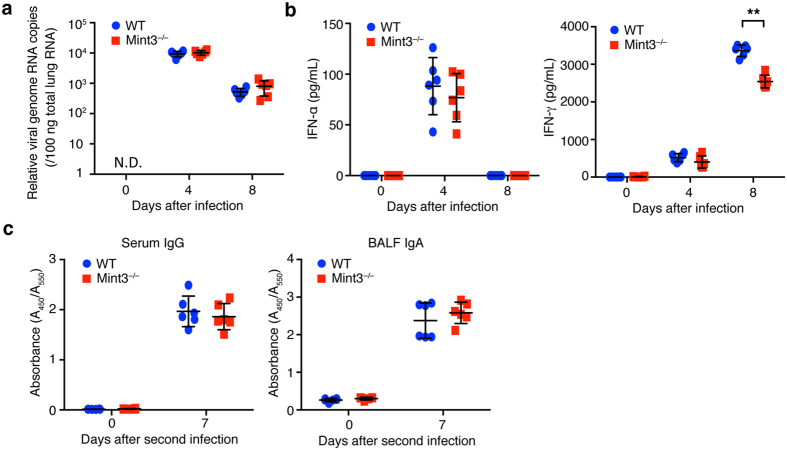
Mint3 deficiency does not affect the induction of type-I interferons and IFV-specific antibody production. (**a**) Viral copy numbers in the infected lungs. The lungs of WT and Mint3^−/−^ mice (n = 6 per group) at day 0, 4, and 8 following infection with IFV were homogenized. Total RNA was extracted from the infected lungs and viral genome RNA copies were quantified by qPCR. (**b**) IFN-α and IFN-γ levels in the BALF from WT and Mint3^−/−^ mice (n = 6 per group) after IFV infection. Data are presented as the means ± SD and are representative of three independent experiments. *P < 0.05, **P < 0.01 by the Student’s t-test. (**c**) IFV-specific antibody production. WT and Mint3^−/−^ mice (n = 6 per group) were intranasally infected twice with 10^3^ PFU of IFV on day 0 and 21. IFV-specific IgG in serum and IgA in the BALF were analysed on day 0 and 7 after the second infection. Data are presented as the means ± SD of triplicates. Data are representative of two independent experiments. *P < 0.05, **P < 0.01 by the Student’s t-test.

Activation of innate immunity in response to IFV regulates anti-viral protective immunity^31^. To evaluate the impact of Mint3 deficiency on anti-IFV protection, we first analysed the viral copy number in the IFV-infected lungs of WT and Mint3^−/−^ mice on day 0, 4, and 8 following the IFV infection. Mint3 depletion did not largely affect the viral copy number in the IFV-infected lungs (Fig. 2a). Type-I interferons (IFN-α/β) are the primary factors that induce host resistance to IFV. In particular, IFN-α induction in the late stage of IFV infection is essential for protection against IFV^31,32^. In contrast to cytokine/chemokine production, no significant difference was observed in IFN-α production in the infected lungs between WT and Mint3^−/−^ mice (Fig. 2b). Notably, the level of the type-II interferon IFN-γ was significantly lower in the lungs of Mint3^−/−^ mice than in those of WT mice on day 8 but not on day 4 after infection (Fig. 2b) for unknown reasons. Next, we assessed the induction of humoral acquired immunity against IFV in Mint3^−/−^ mice. WT and Mint3^−/−^ mice were infected again with IFV on day 21 after the first infection (representing the second infection) and the production of virus-specific antibodies was analysed on day 7 after the second infection. Mint3 depletion in mice affected neither the production of virus-specific IgG in the serum nor virus-specific IgA in the lung mucosa (Fig. 2c). Collectively, these data suggest that Mint3 is dispensable for the induction of protective immunity against IFV infection in mice.

### Mint3 depletion impairs inflammatory cytokine production in response to IFV in macrophages but not dendritic cells

**Figure 3:**
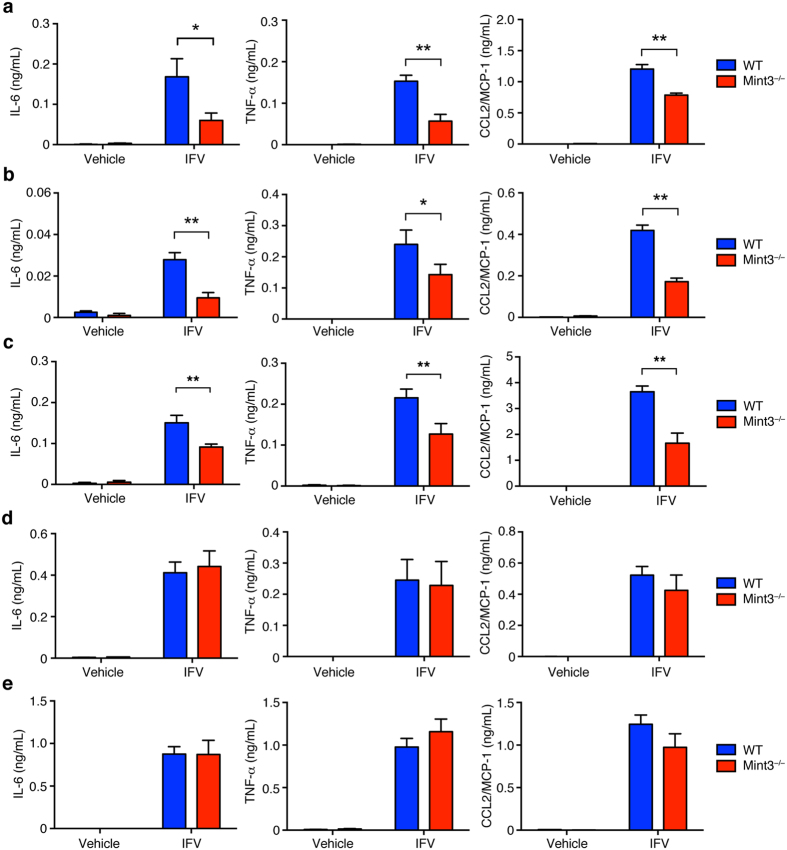
Mint3-mediated signalling controls inflammatory cytokine production in response to IFV in MFs but not DCs. **(a–e)** AMFs (**a**), TG-MFs (**b**), BMMFs (**c**), cDCs (**d**), or FLT3L-DCs (**e**) prepared from WT or Mint3^−/−^ mice were stimulated *in vitro* with 10^6^ PFU IFV (M.O.I = 10) for 24 h. IL-6, TNF-α, and CCL2/MCP-1 production in the cell culture supernatants was measured by ELISA. Data are presented as the means ± SD of triplicates and are representative of two independent experiments. *P < 0.05, **P < 0.01 by the Student’s t-test.

Macrophages and dendritic cells are known to comprise an early source of inflammatory cytokines and type-I interferons in pulmonary IFV infection^11,16^. Mint3 depletion affects macrophage function owing to reduced HIF-1 activity and ATP production via glycolysis^25,27,28^. Thus, we focused on the function of Mint3 in macrophages and dendritic cells, and examined whether Mint3 in macrophages and dendritic cells is required for cytokine/chemokine production in response to IFV. Alveolar macrophages (AMFs), thioglycolate-induced peritoneal macrophages (TG-MFs), bone marrow-derived macrophages (BMMFs), bone marrow-derived conventional dendritic cells (cDCs), or Flt3 ligand-induced plasmacytoid dendritic cells (FLT3L-DCs) prepared from WT or Mint3^−/−^ mice were brought into contact with IFV *in vitro* and the production of IL-6, TNF-α, and CCL2/MCP-1 was measured. The cytokine/chemokine production in response to IFV decreased in Mint3^−/−^ AMFs (Fig. 3a), TG-MFs (Fig. 3b), and BMMFs (Fig. 3c). Furthermore, Mint3 depletion in BMMFs also attenuated cytokine/chemokine production in response to other pathogen-associated molecular patterns (PAMPs) in addition to IFV, such as LPS (Supplementary Fig. S2a), poly I:C (Supplementary Fig. S2b), and imiquimod (R837, Supplementary Fig. S2c). However, Mint3 depletion did not affect IL-6, TNF-α, or CCL2/MCP-1 production in cDCs (Fig. 3d) and FLT3-DCs (Fig. 3e) in response to IFV. Thus, the reduced cytokine/chemokine production by macrophages in response to IFV, but not by dendritic cells, likely accounts at least in part for the attenuation of influenza pneumonia in Mint3^−/−^ mice.

### Mint3 depletion affects NF-κB activation in macrophages

**Figure 4:**
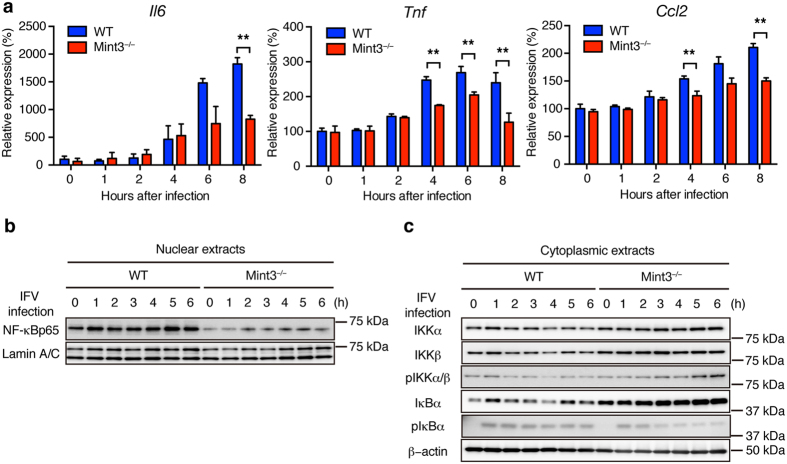
Mint3 depletion affects NF-κB activation in BMMFs. (**a**) *Il6, Tnf*, and *Ccl2* mRNA expression in WT or Mint3^−/−^ BMMFs after IFV infection. (**b**,**c**) NF-κB expression in nuclear extracts (**b**) and NF-κB signalling-related factor expression in cytoplasmic extracts (**c**) of WT or Mint3^−/−^ BMMFs after IFV infection. Data are presented as the means ± SD of triplicates and are representative of two independent experiments. *P < 0.05, **P < 0.01 by the Student’s t-test. Unprocessed original scans of blots are shown in Supplementary Fig. S3.

IFV is recognized by pattern recognition receptors such as Toll-like receptor 7 and retinoic acid inducible gene 1, which then activate the NF-κB signalling pathway in cells^31,33^. Although Mint3^−/−^ macrophages exhibit reduced HIF-1 activity^25,27,28^, it remains unclear whether Mint3 also influences the activation of NF-κB, a transcription factor that induces the expression of inflammatory cytokines/chemokines^34^. Thus, the expression levels of NF-κB target genes such as *Il6, Tnf*, and *Ccl2* in IFV-stimulated WT and Mint3^−/−^ BMMFs were analysed by a quantitative polymerase chain reaction (qPCR) method. The expression levels of *Il6, Tnf*, and *Ccl2* mRNA decreased in Mint3^−/−^ BMMFs following IFV stimulation (Fig. 4a). Next, nuclear translocation of NF-κB in IFV-stimulated BMMFs was examined by immunoblotting. Notably, NF-κB protein levels in the nuclear extracts of Mint3^−/−^ BMMFs were lower than those of WT cells at the starting point (0 h) and remained lower during the observed time after IFV infection (Fig. 4b). Translocation of cytoplasmic NF-κB to the nucleus is usually suppressed by inhibitor of κB (IκB); however, IκB kinase (IKK) phosphorylates IκB and thereby promotes proteasomal degradation of IκB when the NF-κB signalling pathway is activated^34^. Thus, we analysed IκB and IKK in the cytoplasmic extracts from IFV-infected WT and Mint3^−/−^ BMMFs. The expression and phosphorylation levels of IKKα and IKKβ showed no apparent differences between WT and Mint3^−/−^ BMMFs (Fig. 4c). However, the expression levels of IκBα markedly increased in Mint3^−/−^ BMMFs at the starting point (0 h) and remained higher than those of WT cells during the observed time after IFV stimulation (Fig. 4c). In turn, a lower proportion of IκBα was phosphorylated in Mint3^−/−^ BMMFs after IFV stimulation (Fig. 4c). Taken together, these findings indicate that Mint3 depletion resulted in the accumulation of IκBα in the cytoplasm and suppressed the activation of NF-κB in response to IFV stimulation in macrophages.

### Mint3 deficiency affects NF-κB activation in macrophages via both AMPK activation and IκB accumulation

**Figure 5:**
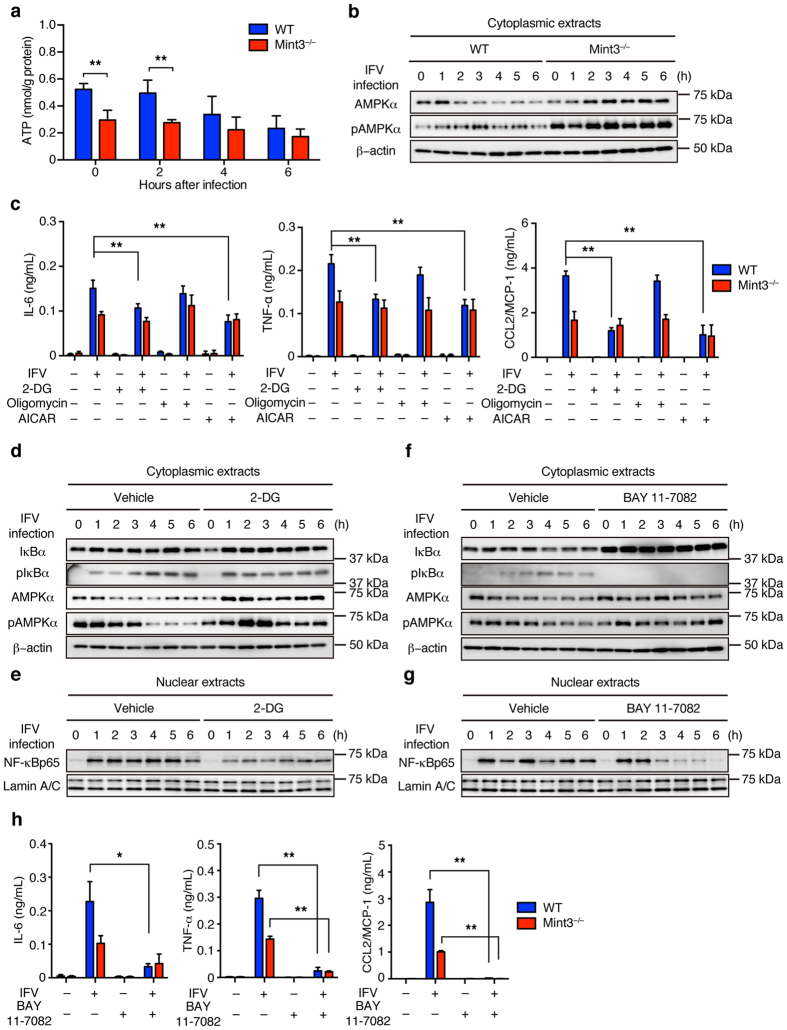
Loss of Mint3 affects NF-κB signalling via both AMPK activation and IκB accumulation in BMMFs. (**a**) ATP content of BMMFs after IFV infection. (**b**) AMPK expression in cellular extracts of WT or Mint3^−/−^ BMMFs after IFV infection. (**c**) BMMFs were pretreated for 1 h with control vehicle (−), 2-DG (100 μg/mL), oligomycin (5 μg/mL), or AICAR (1 mM) and then infected with 10^6^ PFU (M.O.I = 10) IFV for 24 h. IL-6, TNF-α, and CCL2/MCP-1 production in the cell culture supernatants was measured by ELISA. (**d,e**) IκBα and AMPK expression in cytoplasmic extracts (**d**) and NF-κB expression in nuclear extracts (**e**) of WT or 2-DG-treated WT BMMFs after stimulation with IFV. (**f**,**g**) IκBα and AMPK expression in cytoplasmic extracts (**f**) and NF-κB expression in nuclear extracts (**g**) of WT or BAY 11-7082-treated WT BMMFs after stimulation with IFV. (**h**) BMMFs were pretreated for 1 h with control vehicle (−) or BAY 11-7082 (15 μM) and then infected with 10^6^ PFU (M.O.I = 10) IFV for 24 h. IL-6, TNF-α, and CCL2/MCP-1 production in the cell culture supernatants was measured by ELISA. Data are presented as the means ± SD of triplicates and are representative of two independent experiments. **P < 0.05, **P < 0.01 by the Student’s t-test. Unprocessed original scans of blots are shown in Supplementary Fig. S3.

Mint3^−/−^ macrophages exhibit defects in ATP production via glycolysis^25,27,28^. Thus, we next examined whether IFV infection affects ATP levels in BMMFs. ATP levels in WT BMMFs decreased to the levels of Mint3^−/−^ cells 4–6 h after IFV infection, whereas Mint3^−/−^ cells maintained their ATP levels after IFV infection (Fig. 5a). When cells are starved, adenosine monophosphate-activated protein (AMP)-activated kinase (AMPK) is activated by phosphorylation^35^. Accordingly, the phosphorylation levels of AMPKα increased in WT BMMFs after IFV infection (Fig. 5b). Mint3^−/−^ BMMFs showed increased phosphorylation levels of AMPKα at the starting point reflecting their decreased ATP levels (Fig. 5a) and sustained their AMPKα phosphorylation after IFV infection (Fig. 5b). Notably, the amount of AMPKα protein increased after IFV stimulation in Mint3^−/−^ BMMFs (Fig. 5b).

AMPK is known to suppress NF-κB signalling via several pathways such as SIRT1, PGCα1, and p53/FoxO^36^. We hypothesized that the defect of ATP production via glycolysis and the resulting AMPK activation by Mint3 depletion also affects the IFV-induced NF-κB signalling in macrophages. To address this, WT and Mint3^−/−^ BMMFs were infected with IFV in the presence of a glycolysis inhibitor, 2-deoxyglucose (2-DG), a mitochondrial oxidative phosphorylation inhibitor, oligomycin, or vehicle, and the inflammatory cytokine/chemokine production was analysed. 2-DG but not oligomycin treatment decreased the inflammatory cytokine/chemokine production in WT BMMFs to the levels of Mint3^−/−^ cells. The AMPK activator, 5-aminoimidazole-4-carbox-amide-1-β-D-ribofuranoside (AICAR), also decreased the inflammatory cytokine/chemokine production in WT BMMFs to the levels of Mint3^−/−^ cells (Fig. 5c).

Next, we examined whether AMPK activation via the glycolysis defect relates to the IκB accumulation observed in Mint3^−/−^ BMMFs (Fig. 4c). To address this issue, IκBα and AMPKα in IFV-infected WT BMMFs treated with or without 2-DG were analysed. 2-DG treatment did not impact either total or phosphorylated IκBα levels whereas both total and phosphorylated AMPKα levels increased in IFV-infected WT BMMFs (Fig. 5d) and nuclear NF-κB levels decreased (Fig. 5e). These results indicate that AMPK activation via the glycolysis defect attenuates NF-κB signalling independently from IκB accumulation in IFV-infected macrophages. In turn, we next examined whether IκB accumulation alone could attenuate NF-κB signalling in IFV-infected macrophages. To address this possibility, IFV-infected WT BMMFs were treated with or without an IκBα phosphorylation inhibitor, BAY 11-7082^37^. BAY 11-7082 treatment decreased phosphorylated IκBα levels and increased total IκBα levels without affecting total and phosphorylated AMPKα levels in IFV-infected WT BMMFs (Fig. 5f). Under this condition, BAY 11-7082 treatment decreased nuclear NF-κB levels in WT BMMFs 3–6 h after IFV infection (Fig. 5g). BAY 11–7082 treatment also decreased the inflammatory cytokine/chemokine production in WT BMMFs to the levels of Mint3^−/−^ cells (Fig. 5h). Thus, Mint3 depletion independently caused both AMPK activation and IκB accumulation, each of which could separately attenuate NF-κB signalling independently in macrophages.

## Discussion

**Figure 6:**
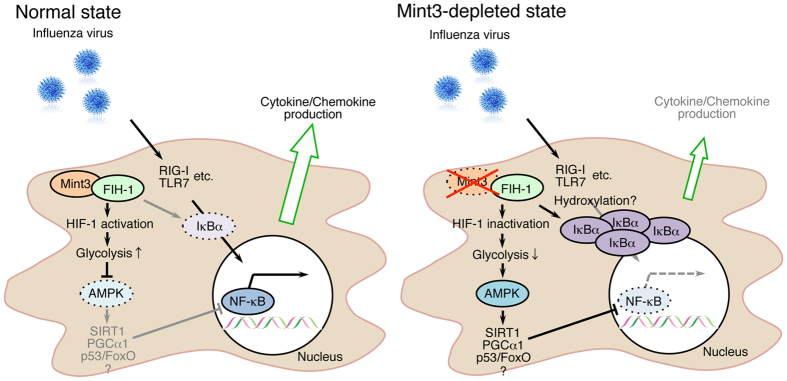
Schematic illustration showing the means by which Mint3 depletion attenuates inflammatory cytokine/chemokine production in IFV-infected macrophages. Mint3 depletion both activates AMPK and promotes IκBα accumulation in macrophages. These mechanisms contribute to inhibiting the nuclear translocation of NF-κB and the resulting inflammatory cytokine/chemokine production in IFV-infected macrophages.

In this study, we have shown that the Mint3-mediated pathway contributes to influenza pneumonia in mice. Mint3 depletion in mice reduced inflammatory cytokine/chemokine production and the infiltration of inflammatory cells in IFV-infected lungs, and improved mortality rates. In macrophages, Mint3 depletion also attenuated cytokine/chemokine production in response to IFV infection by suppressing the NF-κB signalling pathway. This suppression in Mint3^−/−^ macrophages appeared to be mediated by two independent mechanisms: IκBα accumulation and AMPK activation (Fig. 6). Although how AMPK activation suppressed the NF-κB signalling pathway in Mint3^−/−^ macrophages was unclear, several reported pathways such as SIRT1, PGCα1, and p53/FoxO might contribute to this suppression of the NF-κB signalling pathway by AMPK^36^.

Mint3 was considered dispensable for anti-IFV immunity because Mint3 depletion did not alter viral burden, the elevation of IFN-α, or the induction of anti-viral adaptive B cell responses in the IFV-infected mice. Thus, other innate mechanisms independent of the Mint3 pathway may play a more dominant role in protective immunity against IFV. Type II interferon IFN-γ production was reduced in the lungs of Mint3^−/−^ mice compared to that in the lungs of WT mice on day 8 but not on day 4 after IFV infection (Fig. 2b). As IFN-γ expression is regulated by HIF-1^38^, Mint3 depletion might affect IFN-γ expression after IFV infection in mice. IFN-γ is not essential for viral clearance^39^; however, its presence during IFV infection ameliorates the severity of inflammation and lung injury^40^. Although how much the reduction of IFN-γ levels by Mint3 depletion affects the IFV-mediated lung injury remains unclear, supplementation with IFN-γ might support therapies of IFV-infected lung diseases based on Mint3 inhibition.

Mint3 promotes ATP production via glycolysis by activating HIF-1 in macrophages^25,27,28^. AMPK is activated when the ATP concentration decreases in cells^35^. Thus, Mint3 depletion activated AMPK and thereby suppressed the NF-κB signalling in macrophages. Notably, AMPKα protein levels increased after IFV infection in Mint3^−/−^ and 2-DG treated WT macrophages, whereas the basal protein levels of AMPKα were comparable between WT and Mint3^−/−^ macrophages (Fig. 5b,d). Infectious stress might boost the AMPK signalling by increasing the amount of AMPKα protein in macrophages in combination with energy stress. Besides AMPK, HIF-1 also influences cytokine/chemokine transcription directly^41,42^. Thus, Mint3-mediated HIF-1 activation might also contribute to the cytokine/chemokine production independently from AMPK in macrophages. In addition to AMPK, IκBα was also accumulated in Mint3^−/−^ macrophages. Although activated/phosphorylated IKKα/β levels were comparable, less IκBα was phosphorylated in Mint3^−/−^ macrophages than in WT cells after IFV infection. Mint3 activates HIF-1 by suppressing its inhibitor, FIH-1. FIH-1 is an asparaginyl hydroxylase and hydroxylates the α subunit of HIF-1 and other molecules including IκBα^29^. Although the hydroxylation of IκBα by FIH-1 has not been reported to affect the stability of IκBα *in vitro*^43^, FIH-1 might influence the phosphorylation state of IκBα in macrophages.

Mint3-mediated HIF-1 activation depends on an invasion-promoting membrane protease MT1-MMP/MMP14 in macrophages and cancer cells^25,26^. We have recently reported that the monooxygenase NECAB3 and mTOR signalling also promote Mint3-mediated HIF-1 activation in cancer cells^44,45^. Although whether NECAB3 relates to inflammation is unclear, mTOR contributes to inflammatory responses^46,47^. Thus, Mint3 may function as a hub among these molecules and HIF-1, AMPK, and NF-κB signalling pathways to orchestrate inflammation, metabolism, and invasion in macrophages.

In conclusion, Mint3 in macrophages acts to contribute to severe influenza pneumonia, but is dispensable for host protection against IFV. Thus, Mint3 inhibition might represent one of the likely therapeutic targets to control severe IFV infection without affecting viral clearance.

## Materials and Methods

### Mice

Mint3^−/−^ mice have been previously described^27^ (Riken Center for Developmental Biology (CDB) Accession No. CDB0589K; http://www2.clst.riken.jp/arg/mutant%20mice%20list.html). These mice were backcrossed at least 10 times onto C57BL/6 mice. C57BL/6 mice were purchased from CLEA Japan, Inc. (Tokyo, Japan). The animals were housed in specific pathogen-free conditions. All experiments were approved by the Institutional Animal Care and Use Committee for Kitasato University Medical Center and animals were treated in accordance with the Regulations for Animal Experiments in Kitasato University.

### Antibodies and reagents

Fluorescein isothiocyanate (FITC)-conjugated anti-F4/80 (clone BM8), FITC-conjugated CD19 (clone 6D5), phycoerythrin (PE)-conjugated anti-Ly-6G (clone 1A8), biotin-conjugated anti-NK1.1 (clone PK136), peridinin chlorophyll protein/Cy5.5 (PerCP/Cy5.5)-conjugated anti-CD45 (clone 30-F11), and phycoerythrin-Cy7 (PC7)-conjugated anti-CD3ε (clone 145-2C11) monoclonal antibodies were purchased from Biolegend (San Diego, CA). LPS from *Escherichia coli* 0111:B4, poly I:C, and 2-DG were purchased from Sigma-Aldrich (St. Louis, MO). Imiquimod (R837) was purchased from Invivogen (San Diego, CA). Oligomycin was purchased from Merck Millipore (Billerica, MA). AICAR was purchased from Cell Signaling Technology (Danvers, MA). BAY 11-7082 was purchased from Wako Pure Chemical Industries (Osaka, Japan).

### IFV infection in mice

C57BL/6 and Mint3^−/−^ mice were anesthetized and infected with 10^4^ PFU (unless otherwise indicated) of a mouse-adapted IFV (A/California/07/2009 strain: H1N1 isotype, kindly provided by Dr. Takato Odagiri (National Institute of Infectious Diseases, Tokyo, Japan) by intranasal administration as described^30,48,49^.

### Histology

Mice were euthanized by intraperitoneal administration of sodium pentobarbital at 0, 4, and 8 days after IFV infection and whole lungs were collected. Paraffin embedding and hematoxylin and eosin staining of tissues were performed using standard methodologies.

### Bronchoalveolar lavage (BAL)

BAL was carried out as described previously^15,30,48^. In brief, the tracheas of mice were cannulated with 1.2-mm diameter polyethylene catheters. The lungs were instilled with 1 mL pre-warmed phosphate buffered saline (PBS) containing 5 mM ethylenediaminetetraacetic acid, followed by the retrieval of lavage fluid aliquots. Cells in the BAL fluid (BALF) were counted after red blood cell lysis and subjected to flow cytometric analysis. The supernatants of the BALF were subjected to multi cytokine/chemokine expression analysis and enzyme-linked immunosorbent assay (ELISA).

### Flow cytometry

BALF cells were suspended in mouse FcR Blocking Reagent (Miltenyi Biotec, Bergisch Gladbach, Germany) for 10 min prior to staining with FITC-, PE-, biotin-, PerCP/Cy5.5-, or PC7-conjugated antibodies. After staining, biotinylated antibodies were visualized with streptavidin-energy-coupled dye (Beckman Coulter, Fullerton, CA). The antibodies used were anti-F4/80, anti-CD19, anti-Ly-6G, anti-NK1.1, anti-CD45, and anti-CD3ε. Stained cells were analysed with a Cytomics FC500 flow cytometer (Beckman Coulter) and Flowjo software (Tree Star, Ashland, OR).

### Cytokine/chemokine and interferon expression

Cytokine/chemokine and interferon levels in the BALF were measured with a Flow Cytomix Cytokine Bead Assay (Bender MedSystems, Vienna, Austria). Cell culture supernatants from BMMFs were assayed using specific ELISA kits for IL-6, TNF-α (Biolegend), and CCL2/MCP-1 (R&D Systems, Minneapolis, MN). All measurements were performed in triplicate.

### Viral copy numbers

Lung tissues were collected at 0, 4, or 8 days after IFV infection and homogenized using a gentleMACS dissociator (Miltenyi Biotec). RNA was extracted using an Isogen II RNA extraction kit (Nippon Gene, Tokyo, Japan). Reverse transcription was conducted with the Uni-12 primer (5′-AGC AAA AGC AGG-3′)^50^ and qPCR was performed with primers specific for the influenza nucleoprotein gene (*NP*) (Forward: 5′-GAT TGG TGG AAT TGG ACG AT-3′; Reverse: 5′-AGA GCA CCA TTC TCT CTA TT-3′) using the Applied Biosystems 7900HT Fast Real Time PCR System (Life Technologies, Carlsbad, CA). The standard calibration curve for qPCR was obtained by stepwise dilution of the cloned *NP* gene fragment with a known copy number.

### Antigen-specific B cell responses

B cell-mediated humoral responses were measured as virion-specific immunoglobulin production by ELISA, as previously described^51^. Briefly, 96-well ELISA plates (Corning, Corning, NY) were coated with ultrasonicated influenza virion (A/California/07/2009 strain) at 5 × 10^6^ PFU/mL in a carbonate buffer (pH 9.6) and incubated overnight at 4 °C. Plates were then washed with PBS containing 0.05% Tween 20 (Wako). Serum and BALF collected from mice at day 7 after the secondary infection were serially diluted with PBS/Tween 20 containing 5% skim milk, applied onto the virion-coated plates, and incubated for 2 h at room temperature. After washing, goat anti-mouse total IgG or IgA conjugated to horseradish peroxidase (Jackson Immunoresearch, Baltimore Pike, PA) was applied and incubated for 2 h at room temperature. After washing, the plates were stained with a TMB Substrate Set (Biolegend). The reaction was terminated with 1 M H_2_SO_4_ (Wako) and the absorbance was measured.

### MF/DC preparation and IFV stimulation *in vitro*

AMFs were isolated from BALFs of WT or Mint3^−/−^ mice. TG-MFs were prepared as described^52^. BMMFs, cDCs, or FLT3L-DCs were prepared by culturing bone marrow cells for 5–8 days with RPMI1640 medium (Wako) supplemented with 10% foetal bovine serum (Life Technologies) and antibiotics (100 IU/mL penicillin and 100 μg/mL streptomycin; Sigma-Aldrich) containing M-CSF (25 ng/mL, Peprotech, Rocky Hill, NJ), GM-CSF (20 ng/mL, Peprotech), or human Flt3-ligand (100 ng/mL, Peprotech), respectively. For MF/DC stimulation, 1 × 10^5^ cells were seeded on 24-well culture plates (Corning) and incubated overnight, followed by replacement of 200 μL serum-free medium containing 10^6^ PFU (multiplicity of infection [M.O.I] = 10) IFV. After 1 h incubation, the unabsorbed viruses were removed and the cells were incubated for a further 24 h in serum-containing medium. BMMFs were also stimulated with LPS (100 ng/mL), poly I:C (10 μg/mL), or imiquimod (5 μg/mL) for 24 h in serum-containing medium. For chemical reagent treatments, BMMFs were incubated with 2-DG (100 μg/mL), oligomycin (5 μg/mL), AICAR (1 mM) or BAY 11-7082 (15 μM) for 1 h prior to virus stimulation. The culture supernatants were assayed for IL-6, TNF-α, and CCL2/MCP-1 by ELISA.

### qPCR analysis for NF-κB-related mRNAs

Total RNA was extracted from cells using the ReliaPrep RNA Cell Miniprep System (Promega, Madison, WI) and cDNA was synthesized with SuperScript II (Life Technologies) and random primers. The RT products were then subjected to qPCR in a 7500 real-time PCR system (Applied Biosystems, Foster City, CA, USA) using SYBR Green PCR Master Mix (ABI) and specific primers for each gene. The PCR products were sequenced and their homogeneity was confirmed by monitoring the dissociation temperature of SYBR green I fluorescence. Primer sequences were as follows: mouse *Il6*, forward, 5′-TAG TCC TTC CTA CCC CAA TTT CC-3′, and reverse, 5′-TTG GTC CTT AGC CAC TCC TTC-3′; *Tnf*, forward, 5′-AAG CCT GTA GCC CAC GTC GTA-3′, and reverse, 5′-GGC ACC ACT AGT TGG TTG TCT TTG-3′; *Ccl2*, forward, 5′-CCC CAA GAA GGA ATG GGT CC-3′, and reverse, 5′-GGT TGT GGA AAA GGT AGT GG-3′; and *Actb*, forward, 5′-AGA TCA AGA TCA TTG CTC CTC CT-3′, and reverse, 5′-ACG CAG CTC AGT AAC AGT CC-3′.

### Nuclear and cytoplasmic extraction of BMMFs

BMMFs were prepared as described above and stimulated with IFV (M.O.I. = 10). After 1, 2, 3, 4, 5, and 6 h of incubation, the BMMFs were harvested with a cell scraper. Nuclear and cytoplasmic extraction of BMMFs was performed using the Nuclear Extract Kit (Active Motif, Carlsbad, CA) according to manufacturer instruction.

### Immunoblotting

Nuclear and cytoplasmic extracts were separated by sodium dodecyl sulphate-polyacrylamide gel electrophoresis, transferred to membrane filters, and analysed by immunoblotting with a rabbit anti-NF-κBp65 antibody (clone D14E12), a mouse anti-IKKα antibody (clone 3G12), a rabbit anti-IKKβ antibody (clone D30C6), a rabbit anti-phospho-IKKα/β (Ser176/180) antibody (clone 16A6), a mouse anti-IκBα antibody (clone L35A5), a rabbit anti-phospho-IκBα (Ser32) antibody (clone 14D4), a rabbit anti-AMPKα antibody (clone D63G4), a rabbit anti-phospho-AMPKα (Thr172) antibody (clone 40H9) (all obtained from Cell Signaling Technology), a mouse anti-lamin A/C antibody (clone 3A6-4C11, Active Motif), or a mouse anti-β-actin antibody (Merck Millipore).

### Measurement of ATP concentrations

BMMFs were prepared as described above and stimulated with IFV (M.O.I. = 10). After 2, 4, and 6 h of incubation, the BMMFs were harvested with a cell scraper. ATP levels were determined using the ATP bioluminescence assay kit CLS II (Roche Applied Science, Upper Bavaria, Germany) as previously described^26,27^. ATP levels were normalized to the total protein concentration as determined using a Bradford assay kit (Bio-Rad Laboratories, Hercules, CA).

### Statistical analysis

Survival curves were generated by the Kaplan-Meier method and statistical analyses were performed using the log-rank test. The statistical significance was assessed by Student’s t-tests. A *P* value < 0.05 was considered significant.

## Additional Information

**How to cite this article**: Uematsu, T. *et al*. Mint3/Apba3 depletion ameliorates severe murine influenza pneumonia and macrophage cytokine production in response to the influenza virus. *Sci. Rep.* **6**, 37815; doi: 10.1038/srep37815 (2016).

**Publisher's note:** Springer Nature remains neutral with regard to jurisdictional claims in published maps and institutional affiliations.

## Electronic supplementary material

Supplementary Information
