# Supplementary material for: Mint3/Apba3 depletion ameliorates severe murine influenza pneumonia and macrophage cytokine production in response to the influenza virus
Source: Sci Rep. 2016 Nov 24;6:37815. doi: 10.1038/srep37815 (PMC5121658; doi:10.1038/srep37815)
Supplement: Supplementary file 1 — Supplementary Information [file 41598_2016_BFsrep37815_MOESM1_ESM.pdf]

Supplementary Information

Mint3/Apba3 depletion ameliorates severe murine influenza pneumonia and macrophage cytokine production in response to the influenza virus

Takayuki Uematsu[1], Tomoko Fujita[1,2], Hiroki J. Nakaoka[3], Toshiro Hara[4], Noritada Kobayashi[1], Yoshinori Murakami[3], Motoharu Seiki[5] & Takeharu Sakamoto[3]*

[1]Biomedical Laboratory, Department of Biomedical Research, Kitasato University Medical Center, Arai, Kitamoto, Saitama, Japan

[2]Innovation Laboratory, NEC Solution Innovators, Ltd., kashiwanoha, Kashiwa, Chiba, Japan

[3]Division of Molecular Pathology, Institute of Medical Science, The University of Tokyo, Shirokanedai, Minato-ku, Tokyo, Japan.

[4]Division of Cancer Cell Research, Institute of Medical Science, The University of Tokyo, Shirokanedai, Minato-ku, Tokyo, Japan.

[5]Faculty of Medicine, Institute of Medical, Pharmaceutical and Health Sciences, Kanazawa University, Takara-machi, Kanazawa, Ishikawa, Japan.

*Correspondence and requests for materials should be addressed to T.S. (email: t-saka@ims.u-tokyo.ac.jp).

a

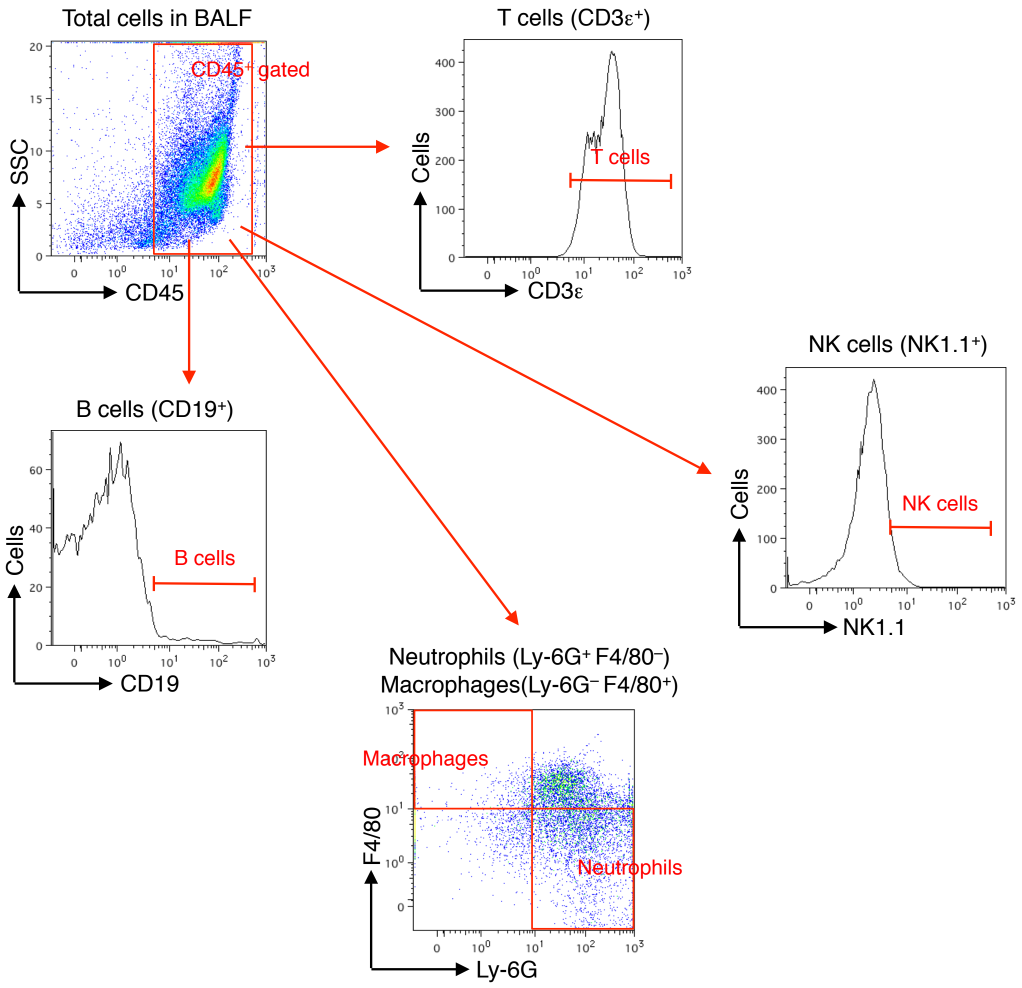

b

Cell number (/mL) = Total cell number in BALF (/mL) × CD45 positive % × { CD3ε positive % (T cells); CD19 positive % (B cells); NK1.1 positive % (NK cells); Ly-6G⁺ F4/80⁻ positive % (Neutrophils); Ly-6G⁻ F4/80⁺ positive % (Macrophages) }

c

【Total cells in BALF】

Days after infection	Mice	Total cell number in BALF (× 10^5/mL)					
		Animal number					
		1	2	3	4	5	6
day0	WT	1.6700	0.8670	0.2670	0.9000	0.7670	0.8000
	Mint3$^{-/-}$	0.8000	1.6700	0.6330	0.9670	0.8330	1.2700
day4	WT	19.5000	15.7000	21.4000	20.8000	17.5000	23.4000
	Mint3$^{-/-}$	13.9000	9.7700	14.5000	11.6000	12.9000	13.5000
day8	WT	45.9000	44.8000	35.7000	30.4000	42.9000	38.7000
	Mint3$^{-/-}$	17.1000	18.7000	22.4000	21.9000	21.7000	19.1000

【T cells】

Days after infection	Mice	T cell number (×10^4/mL)					
		Animal number					
		1	2	3	4	5	6
day0	WT	0.0000	0.0014	0.0017	0.0018	0.0017	0.0009
	Mint3$^{-/-}$	0.0020	0.0020	0.0021	0.0018	0.0023	0.0017
day4	WT	0.5100	0.5100	0.0460	0.5000	0.6100	0.7100
	Mint3$^{-/-}$	0.5700	0.5200	0.4800	0.5100	0.5200	0.5500
day8	WT	1.8400	1.8800	1.8900	1.9100	2.2300	2.1400
	Mint3$^{-/-}$	1.7300	1.7600	1.9100	1.8400	1.8100	2.0200

【B cells】

Days after infection	Mice	B cell number (×10^4/mL)					
		Animal number					
		1	2	3	4	5	6
day0	WT	0.0052	0.0028	0.0033	0.0031	0.0026	0.0016
	Mint3$^{-/-}$	0.0043	0.0046	0.0050	0.0043	0.0045	0.0031
day4	WT	0.0133	0.0119	0.0078	0.0136	0.0116	0.0319
	Mint3$^{-/-}$	0.0113	0.0339	0.0212	0.0177	0.0276	0.0358
day8	WT	0.1470	0.1440	0.0890	0.1500	0.1470	0.0680
	Mint3$^{-/-}$	0.1303	0.1317	0.0619	0.0633	0.0468	0.1625

【NK cells】

Days after infection	Mice	NK cell number (×10^4/mL)					
		Animal number					
		1	2	3	4	5	6
day0	WT	0.0019	0.0047	0.0055	0.0064	0.0046	0.0015
	Mint3$^{-/-}$	0.0057	0.0057	0.0067	0.0062	0.0061	0.0021
day4	WT	0.2231	0.2340	0.2231	0.2234	0.2259	0.2897
	Mint3$^{-/-}$	0.2779	0.2566	0.2480	0.2432	0.2564	0.1987
day8	WT	0.1974	0.2030	0.1745	0.1891	0.1910	0.2368
	Mint3$^{-/-}$	0.2339	0.2230	0.2392	0.2234	0.2298	0.1854

【Neutrophils】

Days after infection	Mice	Neutrophil number (×10^4/mL)					
		Animal number					
		1	2	3	4	5	6
day0	WT	0.0006	0.0010	0.0011	0.0012	0.0010	0.0025
	Mint3$^{-/-}$	0.0018	0.0018	0.0018	0.0016	0.0017	0.0005
day4	WT	17.0100	16.2020	23.6230	15.8820	15.6792	19.2365
	Mint3$^{-/-}$	14.3710	11.6730	10.8160	7.1550	13.5037	9.8198
day8	WT	22.5640	32.7900	19.6960	22.6740	24.4310	27.8925
	Mint3$^{-/-}$	15.6650	12.7480	14.4450	17.7230	17.6452	13.2624

【Macrophages】

Days after infection	Mice	Macrophage number (×10^4/mL)					
		Animal number					
		1	2	3	4	5	6
day0	WT	0.0015	0.0024	0.0029	0.0027	0.0020	0.0011
	Mint3$^{-/-}$	0.0023	0.0025	0.0012	0.0026	0.0019	0.0321
day4	WT	0.7290	0.7219	0.7690	0.7693	0.7473	0.5287
	Mint3$^{-/-}$	0.7769	0.6440	0.8652	0.5363	0.8306	0.7258
day8	WT	0.8806	0.6577	0.4908	0.5874	0.6791	0.6514
	Mint3$^{-/-}$	0.7370	0.4643	0.7190	0.5260	0.4365	0.6523

Supplementary Figure S1

FACS analytical methods in main Figure 1c.

(**a**) FACS gating strategies of total cells in BALF. The plot data are representative ones. (**b**) Calculation methods of the absolute cell numbers of T cells, B cells, NK cells, neutrophils, and macrophages. (**c**) The raw data of the absolute cell numbers of T cells, B cells, NK cells, neutrophils, and macrophages in main Figure 1c.

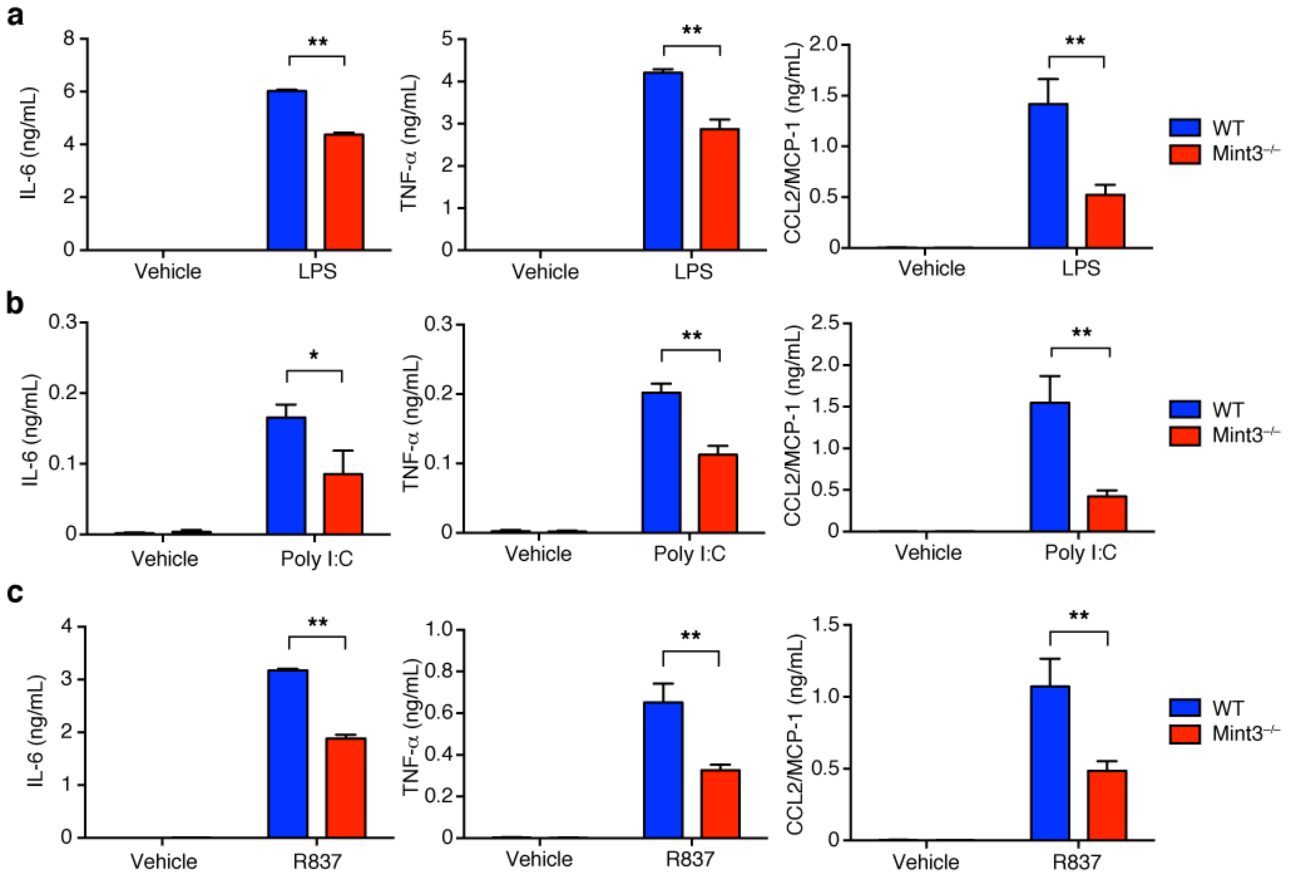

Supplementary Figure S2

Mint3-mediated signaling controls inflammatory cytokine production in response to PAMPs by BMMFs.

BMMFs prepared from WT or Mint3$^{-/-}$ mice were stimulated *in vitro* with (**a**) LPS (100ng/mL), (**b**) poly I:C (10μg/mL), or (**c**) imiquimod (R837, 5μg/mL) for 24 h. IL-6, TNF-α and CCL2/MCP-1 production in the cell culture supernatants were measured by ELISA. Data are presented as mean ± SD of triplicates, and are representative of two independent experiments. **P*<0.05, ***P*<0.01 by Student's t-test.

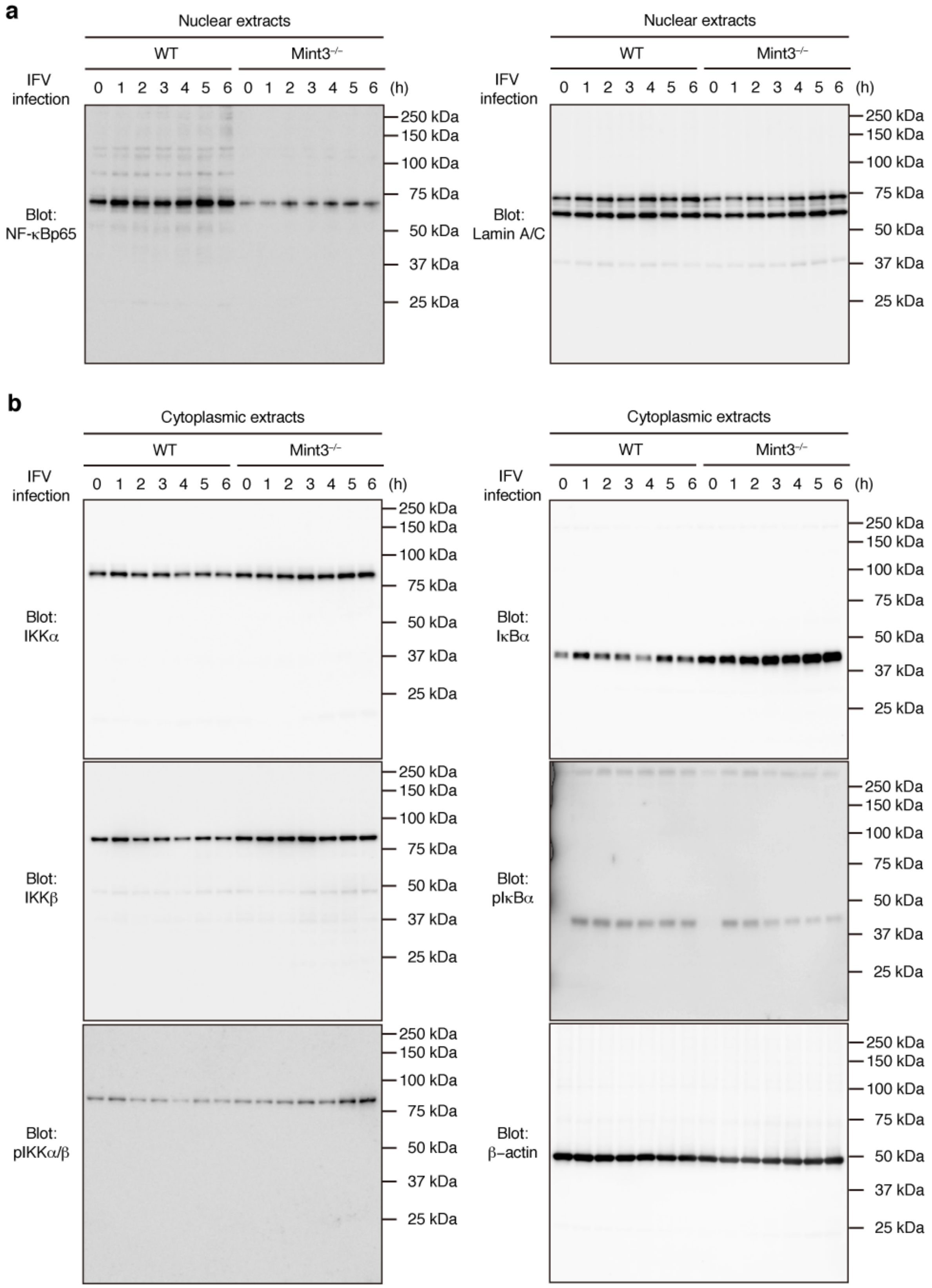
a
Nuclear extracts
WT
Mint3−/−
IFV infection
0 1 2 3 4 5 6 0 1 2 3 4 5 6 (h)
250 kDa
150 kDa
100 kDa
75 kDa
50 kDa
37 kDa
25 kDa
Blot: NF-κBp65
Blot: Lamin A/C
b
Cytoplasmic extracts
Blot: IKKα
Blot: IKKβ
Blot: pIKKα/β
Blot: IκBα
Blot: pIκBα
Blot: β-actin

c

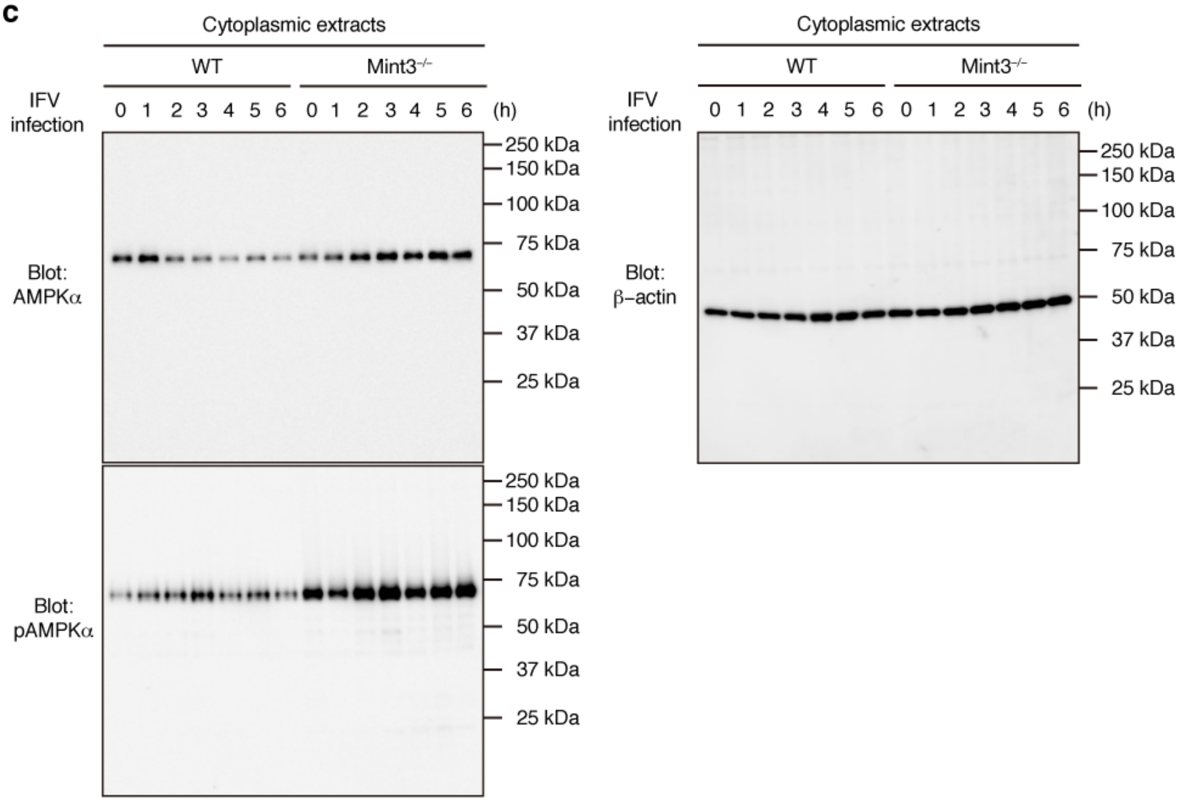

Cytoplasmic extracts
WT
Mint3−/−
IFV infection
0 1 2 3 4 5 6 0 1 2 3 4 5 6 (h)
Blot: AMPKα
Blot: pAMPKα
Blot: β-actin
250 kDa
150 kDa
100 kDa
75 kDa
50 kDa
37 kDa
25 kDa

d

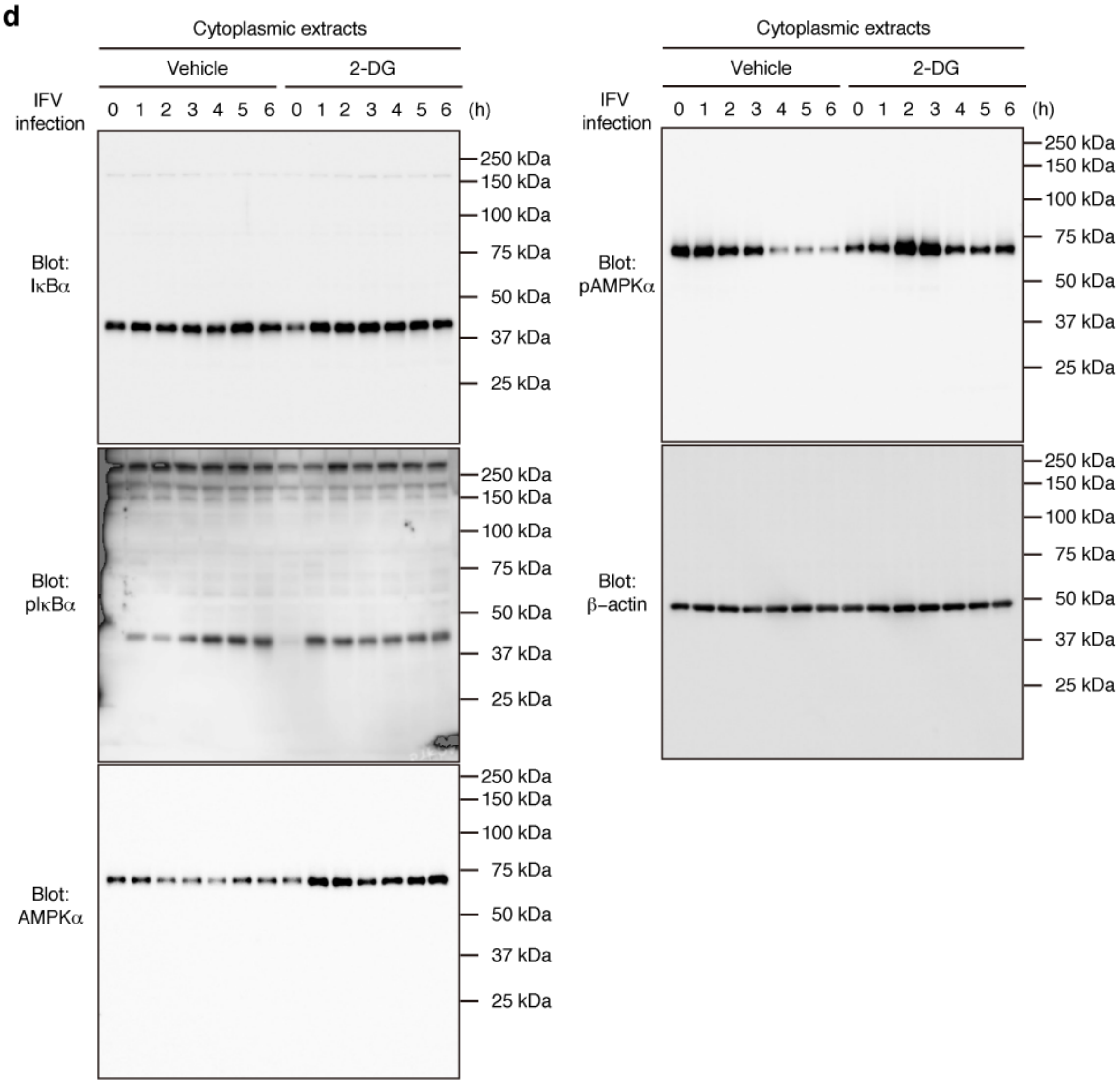
Cytoplasmic extracts
Vehicle
2-DG
IFV infection
0 1 2 3 4 5 6 0 1 2 3 4 5 6 (h)
Blot: IκBα
Blot: pIκBα
Blot: AMPKα
Blot: pAMPKα
Blot: β–actin
250 kDa
150 kDa
100 kDa
75 kDa
50 kDa
37 kDa
25 kDa

e

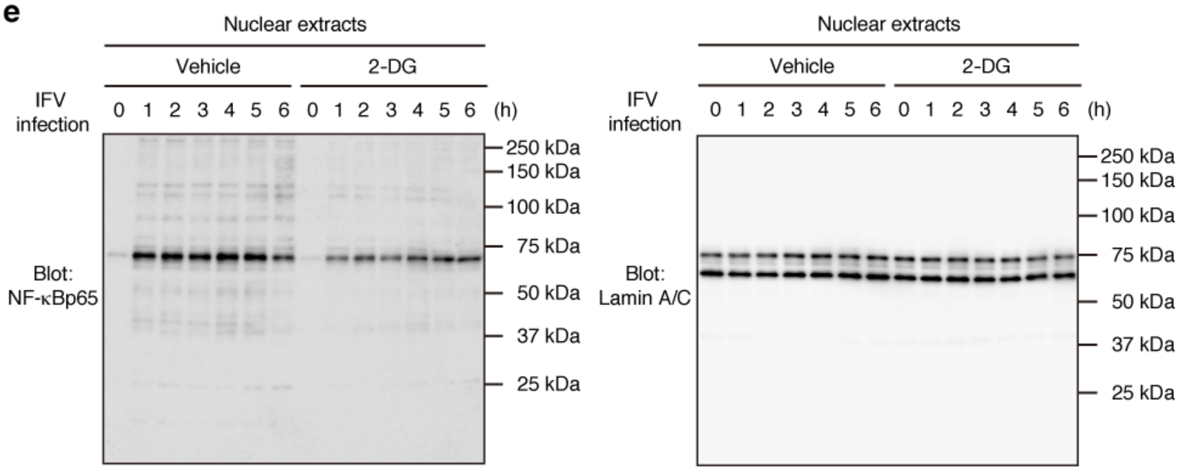
Nuclear extracts
Vehicle
2-DG
IFV infection
0 1 2 3 4 5 6 0 1 2 3 4 5 6 (h)
Blot: NF-κBp65
Blot: Lamin A/C
250 kDa
150 kDa
100 kDa
75 kDa
50 kDa
37 kDa
25 kDa

f

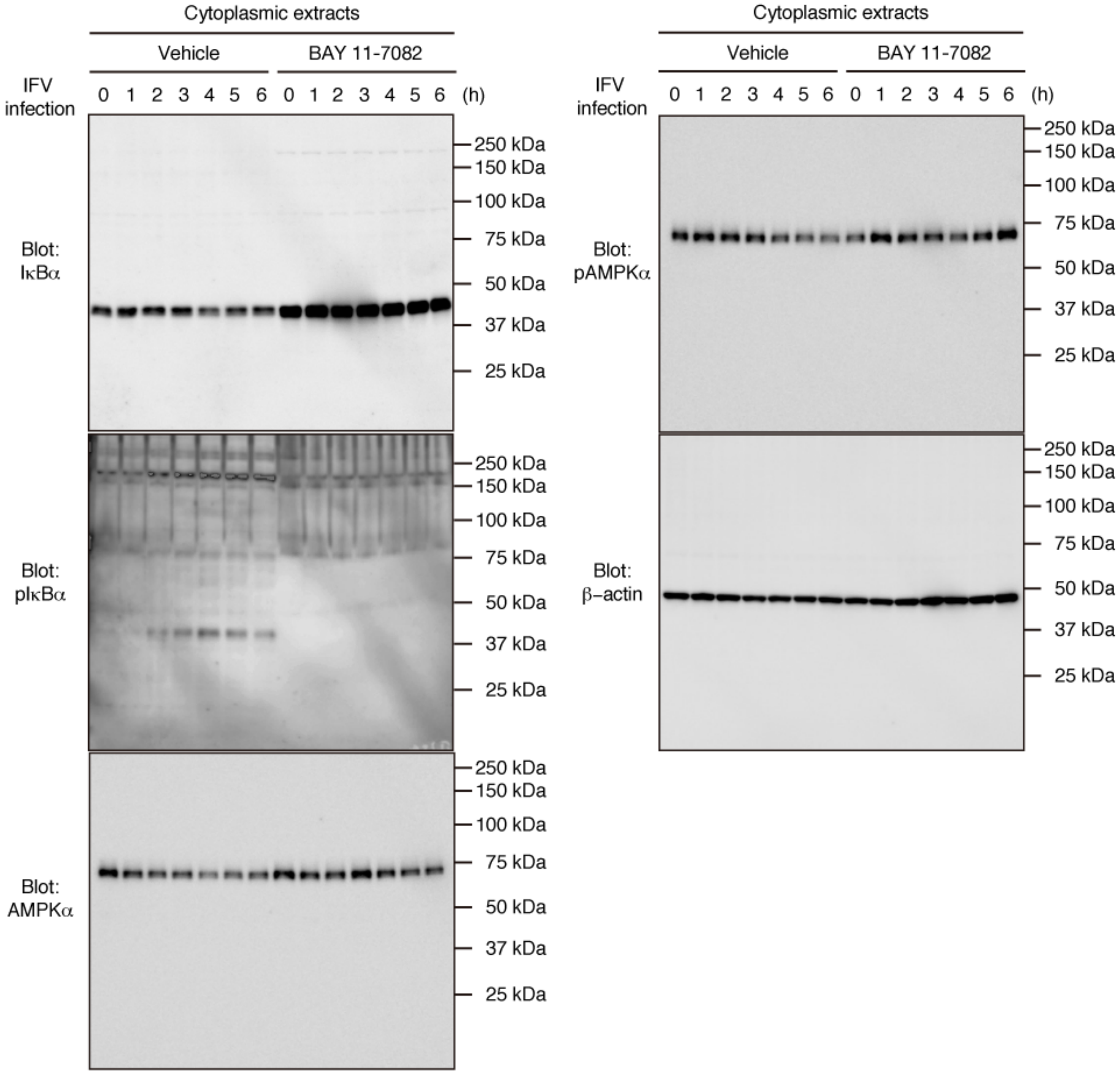

g

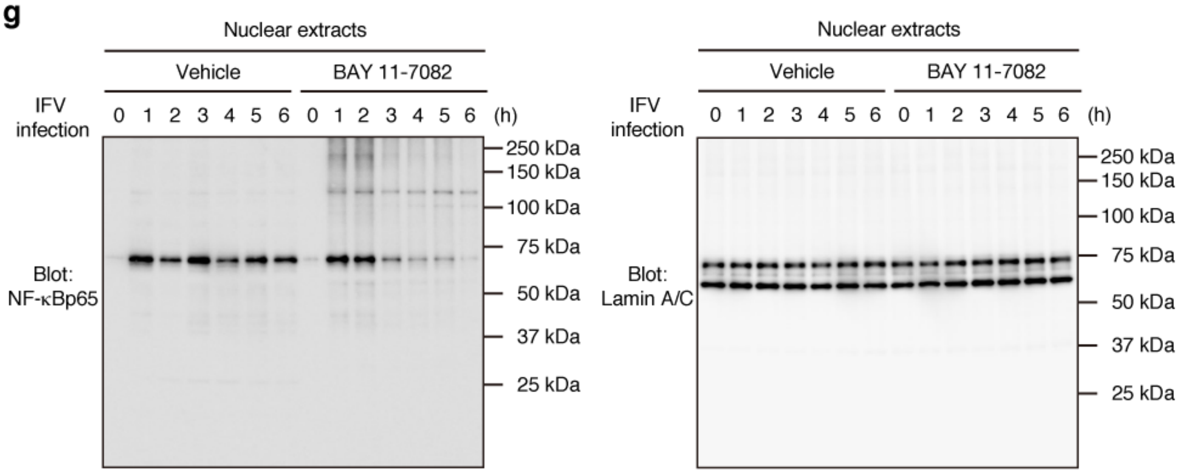

Supplementary Figure S3

Full scans of Immunoblots in the main figures.

(**a**) Full blots of *Figure 4b*. (**b**) Full blots of *Figure 4c*. (**c**) Full blots of *Figure 5b*. (**d**) Full blots of *Figure 5d*. (**e**) Full blots of *Figure 5e*. (**f**) Full blots of *Figure 5f*. (**g**) Full blots of *Figure 5g*.
